# Supplementary material for: Empirical methods for controlling false positives and estimating confidence in ChIP-Seq peaks
Source: BMC Bioinformatics. 2008 Dec 5;9:523. doi: 10.1186/1471-2105-9-523 (PMC2628906; doi:10.1186/1471-2105-9-523)
Supplement: Additional File 1 — A variety of html documents from the USeq web site detailing the available applications, their best usage, output file type descriptions, command line menus, etc. [file 1471-2105-9-523-S1.zip › USeqUserGuides/JavaDoc.zip!/JavaDoc/edu/utah/seq/methylation454/DataLine.html]

DataLine


|  |  |  |
| --- | --- | --- |
| **Overview** \| **Package** \| **Class** \| **Use** \| **Tree** \| **Deprecated** \| **Index** \| **Help** | |  |
| **PREV CLASS**   **NEXT CLASS** | **FRAMES**    **NO FRAMES**     **All Classes** |  |
| SUMMARY: NESTED \| FIELD \| CONSTR \| METHOD | DETAIL: FIELD \| CONSTR \| METHOD |  |


---


## edu.utah.seq.methylation454 Class DataLine

```
java.lang.Object
  edu.utah.seq.methylation454.DataLine
```

**All Implemented Interfaces:**: java.lang.Comparable

---

``` public class DataLine extends java.lang.Object implements java.lang.Comparable ```

---

**Constructor Summary**

`DataLine(java.lang.String line)`


| **Method Summary** | |
| --- | --- |
| `int` | `compareTo(java.lang.Object obj)` |
| `java.lang.String` | `getAmplicon()` |
| `java.lang.String` | `getCpG()` |
| `java.lang.String` | `getRead()` |
| `java.lang.String` | `getSample()` |
| `java.lang.String` | `getSequence()` |
| `java.lang.String` | `getStrand()` |
| `java.lang.String` | `toString()` |

**Methods inherited from class java.lang.Object**

`equals, getClass, hashCode, notify, notifyAll, wait, wait, wait`

**Constructor Detail**

### DataLine

```
public DataLine(java.lang.String line)
```


**Method Detail**

### toString

```
public java.lang.String toString()
```

:   **Overrides:**: `toString` in class `java.lang.Object`

---


### compareTo

```
public int compareTo(java.lang.Object obj)
```

:   **Specified by:**: `compareTo` in interface `java.lang.Comparable`

---


### getAmplicon

```
public java.lang.String getAmplicon()
```

---


### getCpG

```
public java.lang.String getCpG()
```

---


### getRead

```
public java.lang.String getRead()
```

---


### getSample

```
public java.lang.String getSample()
```

---


### getSequence

```
public java.lang.String getSequence()
```

---


### getStrand

```
public java.lang.String getStrand()
```


---


|  |  |  |
| --- | --- | --- |
| **Overview** \| **Package** \| **Class** \| **Use** \| **Tree** \| **Deprecated** \| **Index** \| **Help** | |  |
| **PREV CLASS**   **NEXT CLASS** | **FRAMES**    **NO FRAMES**     **All Classes** |  |
| SUMMARY: NESTED \| FIELD \| CONSTR \| METHOD | DETAIL: FIELD \| CONSTR \| METHOD |  |


---
